# Strongest Correlation Between Contrast Sensitivity and Morphological Characteristics in Bilateral nAMD

**DOI:** 10.3389/fmed.2020.622877

**Published:** 2021-01-28

**Authors:** Laura Hoffmann, Petra Rossouw, Maria-Magdalena Guichard, Katja Hatz

**Affiliations:** ^1^Vista Klinik, Binningen, Switzerland; ^2^Department of Vision Science and Optometry, University of Aalen, Aalen, Germany; ^3^Faculty of Medicine, University of Basel, Basel, Switzerland

**Keywords:** contrast sensitivity, age-reated macular degeneration, AMD, visual function, OCT, OCTA, geographic atrophy, fundus autofluorecence

## Abstract

In patients with neovascular age-related macular degeneration (nAMD) there is often an inconsistency between their subjective visual impairment and a still relatively preserved standard Early Treatment of Diabetic Retinopathy Study (ETDRS) best corrected visual acuity. Therefore, in order to better capture the specific functional defects in nAMD, other tests need to be evaluated. In a previous study, we reported contrast sensitivity of the better eye to best correlate with near distance and distance vision related quality of life in patients with bilateral nAMD. Here, we evaluated Pelli-Robson contrast sensitivity, ETDRS visual acuity, low luminance visual acuity and Radner maximum reading speed and correlated them with several morphologic parameters as measured on fundus autofluorescence imaging, optical coherence tomography and optical tomography angiography in 54 patients. A multiple regression analysis was performed which correlated each visual function parameter with the anatomic features. The results showed the strongest correlations between the total area of macular geographic atrophy as well as the percentage of geographic atrophy in the central 1 mm and contrast sensitivity. Further, the regression model selected the total area of macular geographic atrophy, the photoreceptor inner and outer segments interface disruption score, the presence of subretinal fibrosis in the central 1 mm and the central retinal thickness as the variables that explained 71% of the variation in contrast sensitivity when including all eyes. Hence, our results suggest that among the evaluated measures of vision, contrast sensitivity is best correlated with the morphologic impairment in bilateral nAMD. Thus, contrast sensitivity may complement ETDRS visual acuity in clinical trials and serve as a standard diagnostic tool in clinical practice.

## Introduction

Age-related macular degeneration (AMD) represents the leading cause of blindness in the elderly in the industrialized world and a major public health concern (1). As a result of the current demographic development, a considerable rise in the affected subjects is estimated in the future (2). Despite typically mild visual impairment in the early stages, the progression of the disease results in a severe central visual loss, which causes disability especially when both eyes are involved (3, 4). Progressive AMD is either represented by macular neovascularization (MNV) or an advanced stage of dry AMD mainly characterized by geographic atrophy (GA) of the outer retina and the retinal pigment epithelium (RPE) which might initially spare the fovea (5, 6). Eyes with neovascular AMD (nAMD) sometimes show GA zones at diagnosis or develop GA as well as subretinal scarring of MNV over the long-term (7). Subretinal fibrosis and GA, both leading to defects in the outer retina, account for the principal causes of vision loss in nAMD (8).

Typically, while patches of atrophy primarily present in the parafovea leading to difficulties in near-distance vision-related tasks such as reading, the central distance visual acuity [standard Early Treatment of Diabetic Retinopathy Study (ETDRS) protocol measurement at 4 m] is still relatively preserved (9). The loss of the RPE results in the decline of the photoreceptors so that the areas of GA correspond to an absolute scotoma (10). Within those lesions and, to a lesser extent in the still functional retina, a reduction in retinal sensitivity can be measured by the use of microperimetry (11, 12). In addition, with the enlargement of GA, a decrease in retinal sensitivity occurs (6, 13). The areas of reduced retinal sensitivity correlate morphologically with an impairment of the outer retina as observed by Optical Coherence Tomography (OCT), namely a disruption of the photoreceptors' inner and outer segments (IS/OS) interface and loss of the external limiting membrane (ELM) (14–16).

However, in AMD an inconsistency exists between the severity of the morphologic impairment and visual function since a wide range of visual acuity is observed in clinical practice (17). Furthermore, conventional high-contrast best-corrected visual acuity (BCVA) testing (ETDRS at 4 m), which is widely used in clinical practice and trials, does not adequately correlate with the patient's ability to execute vision-related tasks under conditions of everyday life. Even in the early stages, patients experience difficulties under low lightning and low contrast conditions, while central distance visual acuity is still reasonably intact (18). Hence, other functional tests have been evaluated to better correlate with the subjective visual impairment. Contrast sensitivity (CS) and low-luminance visual acuity (LLVA) have been reported to best capture the specific functional impairment in AMD when compared to healthy controls (19, 20). For our study population, contrast sensitivity is also documented to strongest correlate with patient's vision-related quality of life as measured by the National Eye Institute Visual Function Questionnaire (NEI-VFQ25) (21).

These findings suggest to evaluate whether alternative functional visual measures might be more reflective of macular morphology than conventional BCVA testing. Several quantitative and qualitative morphologic parameters, namely the retinal thickness, subretinal fibrosis and location of GA have been shown to correlate with standard distance visual acuity (15, 22–24). Furthermore, pre-existing RPE atrophy and disruption in the ELM are described to serve as predictors for an early poor visual response to anti-vascular endothelial growth factor (anti-VEGF) treatment in nAMD (7). Due to the heterogeneity of the morphologic impairment in AMD, data suggest that a combination of factors including location, size and component of the lesions explain the variety of visual function in AMD (23, 25). Currently, there are few studies describing the influence of foveal involvement of fibrosis and GA and size and location of MNV on contrast sensitivity which, in addition, did not use the latest image acquisition devices (23, 26, 27).

Amendments in OCT technology reveal detailed insight in outer retinal layer changes in eyes with AMD (28). Nevertheless, there are currently limited data correlating systemically alternative visual testing parameters and the plenitude of morphologic parameters as measured by fundus autofluorescence (FAF), spectral domain OCT (SD-OCT) and swept source OCT Angiography (SS-OCT-A). Therefore, the aim of our study was to evaluate the relations between different measures of visual function (distance acuity, contrast sensitivity, reading speed and LLVA) and morphologic characteristics in patients with bilateral nAMD to identify validated morphologic parameters which may serve as endpoints in clinical practice and research.

## Patients and Methods

This was a cross-sectional, non-interventional and single-visit study of patients treated at the Vista Klinik, Binningen, Switzerland with a clinically confirmed diagnosis of bilateral nAMD by a retina specialist. The study received approval from the local ethics approval board [Ethikkommission Nordwestschweiz (EKNZ No. 2016-002216)] and was performed in accordance with the tenets of the Declaration of Helsinki and Good Clinical Practice (ICH-GCP). The study is registered at ClinicalTrial.gov (NCT 03438669).

Data were obtained between February 2017 and October 2017. Patients had to meet the following inclusion criteria to be eligible: age ≥ 55 years, bilateral nAMD with AMD-related lesions such as MNV or GA or subretinal fibrosis or pigment epithelial detachment (PED) within the central 1 mm ETDRS grid subfield confirmed by Spectral Domain Optical Coherence Tomography (SD-OCT; Spectralis, Heidelberg Eng., Heidelberg, Germany) and Swept-Source-OCT-Angiography (SS-OCTA; Plex Elite 9000, Carl-Zeiss Meditec, Dublin, USA), best corrected visual acuity (BCVA) of at least 49 letters (Snellen equivalent 20/100 or better) in the better eye using ETDRS charts at a distance of 4 m, sufficiently clear ocular media and adequate pupillary dilation and fixation permitting quality fundus imaging. All eyes were currently treated with intravitreal anti-vascular endothelial growth factor (VEGF) agents. Exclusion criteria were significant ocular disease other than neovascular AMD or a history of neurologic disease or cognitive impairment.

### Visual Function Assessment

Study examinations took place no earlier than 4 days after the last intravitreal treatment and were always performed in the same order. All patients underwent a standardized refraction protocol with ETDRS BCVA at 4 m in designated rooms with an ambient illumination of 97–109 lux evaluated with a calibrated illuminometer. LLVA testing was realized immediately afterwards following the LLVA testing protocol of our clinic. First light switches were turned off and then the block out shutters were closed in order to have the ETDRS light box as the only illumination left. LLVA testing took place once the patients were ready to continue and no difficulties adapting to the lower lighting conditions were reported. Since the testing was not always performed in the same rooms because of differing vacancies and time of appointment, the illumination varied slightly between 02 and 10l ux. The low luminance deficit (LLD) was defined as the number of ETDRS letters read in standard BCVA testing minus LLVA. Maximal reading speed was tested monocularly and binocularly with Radner reading charts (Precision Vision, Inc.). Contrast sensitivity was measured using Pelli Robson charts (Precision Vision, Inc.) at 1 m. Further details of the visual function assessment in this study are described elsewhere (21).

### Retinal Imaging Analysis

Morphological impairment was evaluated with SD-OCT (Heidelberg Engineering, Germany), using the following scans: horizontal volume scan 19 sections, macular star 9 sections and horizontal 6 mm scan. Grading was performed by two masked physicians according to a standardized protocol (LH, MG). The foveola was localized in the volume scans to overlay an ETDRS grid centered on the fovea. All lesions seen on OCT, FAF and OCT-A scans were classified dichotomously depending on presence or absence in each of the nine subfields of the disposed ETDRS grid. Lesions were then summarized as located within the central 1, 3, or 6 mm ring.

Photoreceptor inner segment and outer segments (IS/OS) interface and external limiting membrane (ELM) disruption were evaluated by calculating the mean of the score in the horizontal and vertical scans of the macular star scan (29). Disruption was graded as 0 (no disruption in 1 mm center), 1 (mild disruption <1/4 in 1 mm center), 2 (1/4 to 3/4 disruption in 1 mm center) and 3 (>3/4 disruption in 1 mm center) (29). Central subfield thickness (CRT) analysis was performed after centering of the scan and manual alignment of the automated lines. All scans were graded with regard to the presence of subretinal fibrosis and pigment epithelial detachment (PED). Subretinal fibrosis was considered as subretinal hyper-reflective material at the level above the retinal pigment epithelium. PED was defined as a focal elevation of the retinal pigment epithelium presenting with a height ≥200 μm or a width ≥400 μm (30). Maximum height of pigment epithelial detachment in the central 1 mm was measured.

Geographic atrophy (GA) was assessed by fundus autofluorescence imaging acquired with confocal scanning laser ophthalmoscope (HRA, Heidelberg Engineering, Germany) with an excitation wavelength of 488 nm and an emission spectrum of 500–700 nm. The GA pattern was classified as focal or multifocal and the surface area was measured with the built-in free-hand draw tool. Percentage of the spared surface within the central 1 mm ring was calculated.

All OCT-A analyses were performed with PLEX Elite 9000 Swept-Source OCT Angiography (Carl Zeiss Meditec Inc, Dublin, USA) using a 6 mm scan centered on the fovea. Manual correction was carried out to ensure an accurate segmentation. The CNV membrane was located in the en face OCT-A slabs of the outer retinal layer and choriocapillaris (ORCC) and surface was measured using the Image J tool (NIH Image, Bethesda, MD).

### Statistical Analysis

Statistical analysis was performed using SPSS statistical package version 21 (SPSS, Inc., Chicago, IL). Data are presented as mean ± standard deviation (SD) or percentages, as appropriate. Lesions were subdivided depending on their presence or absence in the central 1, 3, or 6 mm ring and an unpaired *t*-test for comparison of means was used to test for differences in visual function between these groups. *P*-values for multiple comparisons were adjusted according to the Bonferroni-Holm adjustment (31). To evaluate correlations between morphologic and visual function parameters, Pearson correlation coefficients were calculated. Afterwards a multiple regression analysis was performed using the variables which were previously statistically significantly correlated with visual function as explanatory variables. To reduce potential collinearity, highly correlated variables (*r* > 0.90) were not included in the same model. Backward stepwise elimination was performed to select independent explanatory parameters. Each time a visual function parameter was used as the dependant variable and the model with the highest coefficient of determination (R^2^) was chosen. Despite the known dependence of both eyes of a patient (31), here data are reported for the better, the worse and for both eyes of each patient nevertheless in order to evaluate a possible early biomarker reflective of retinal morphology when comparing the correlations with retinal morphology according to the status of the eye. *P*-values <0.05 were considered statistically significant.

## Results

### Patient Characteristics

This study included 108 eyes from 54 patients with a mean age of 79.7 ± 7.8 years. 53.7% of patients were female, 46.3% were male.

### Visual Function

Detailed analysis of the assessment of visual function of the study population has been described elsewhere (21). Mean BCVA for all eyes was 66.75 ± 24.33 ETDRS letters (0.37 ± 1.21 logMAR) with a statistically significant difference between the better and the worse eye (78.91 ± 7.93 letters (0.12 ± 1.54 logMAR) and 47.43 ± 35.96 letters (0.75 ± 0.98 logMAR), respectively, *p* < 0.001), see Table 1. Mean LLVA showed comparable results with a mean of 66.06 ± 24.17 letters (0.38 ± 1.22 logMAR). Mean contrast sensitivity for all eyes was 1.21 ± 0.42 log units. Mean binocular maximum reading speed did not differ significantly from mean maximum reading speed of the better eye (*p* = 0.7287). Mean monocular maximum reading speed for all eyes was significantly lower than binocular maximum reading speed (96.87 ± 48.16 wpm, *p* < 0.001).

**Table 1:** Visual assessment.

	**All eyes (*n* = 108)**	**Better eyes (*n* = 54)**	**Worse eyes (*n* = 54)**
Mean BCVA (ETDRS letters) (logMAR) ±SD	66.75 ± 24.33 (0.37 ± 1.21)	78.91 ± 7.93 (0.12 ± 1.54)	47.43 ± 35.96 (0.75 ± 0.98)
Mean LLVA (ETDRS letters) (logMAR) ±SD	66.06 ± 24.17 (0.38 ± 1.22)	78.13 ± 8.04 (0.14 ± 1.54)	47.37 ± 35.39 (0.75 ± 0.99)
Low luminance deficit (LLD in letters)	0.69 ± 3.41	0.98 ± 3.81	0.41 ± 2.94
Mean CS (log units) ±SD	1.21 ± 0.42	1.33 ± 0.27	1.09 ± 0.50
Mean monocular MRS (wpm) ±SD	96.87 ± 48.16	118.16 ± 27.88	75.59 ± 54.61
Mean binocular MRS (wpm) ±SD	117.33 ± 28.42		
NEI-VFQ25 near distance subscale	74.69 ± 18.74		
NEI-VFQ25 distance subscale	74.15 ± 21.90		

### Morphologic Parameters

Morphological characteristics are summarized in Table 2. There was a statistically significant difference for all of these parameters regarding the status of the eyes (better vs. worse eyes, *p* < 0.01) except for mean total area of CNV, presence of PED in the central 1 mm and disruption of the IS/OS interface in the central 1 mm. 26.9% of all eyes (*n* = 29) presented without geographic atrophy. For all other eyes, mean area of total macular GA was 3.02 ± 6.67 mm^2^, mean percentage of GA in the central 1 mm was 19.40 ± 32.99%. Considering only the eyes with GA (*n* = 79), 49.4% presented with multifocal GA. 63.9% of all eyes (*n* = 69) had sparing in the central 1 mm.

**Table 2:** Morphologic characteristics.

	**All eyes (*n* = 108)**	**Better eyes (*n* = 54)**	**Worse eyes (*n* = 54)**
**FAF**			
Mean total area of GA (mm^2^) ±SD	3.02 ± 6.67	1.41 ± 3.90	4.60 ± 8.30
Mean percentage of GA in central 1 mm ±SD	19.40 ± 32.99	9.76 ± 23.95	29.05 ± 37.86
**OCT**			
Mean CRT (μm) ±SD	332.60 ± 187.45	294.39 ± 76.56	353.09 ± 201.94
Disruption IS/OS interface central 1 mm (%)	91.7	90.7	92.6
Disruption ELM central 1 mm (%)	83.3	81.5	85.2
Subretinal fibrosis central 1 mm (%)	17.6	3.7	31.5
PED in central 1mm (%)	59.3	66.7	51.9
**OCT-A**			
Mean total area of CNV (mm^2^) ±SD	2.10 ± 3.23	1.53 ± 1.77	2.69 ± 4.17

Mean central retinal thickness (CRT) was 332.60 ± 187.45 μm, mean height of pigment epithelium detachment (PED) in the central 1 mm was 156.02 ± 103.41 μm. 8.3% of all eyes (*n* = 9) showed an intact junction of the photoreceptors inner and outer segments (IS/OS junction line) and 16.7% (*n* = 18) an intact external limiting membrane (ELM). 17.6% of all eyes (*n* = 19) presented with subretinal fibrosis and 59.3% (*n* = 64) with a PED in the central 1 mm. 25 eyes (23.1%) presented with reticular pseudodrusen (RPD). 82.4% of all eyes (*n* = 89) showed a clearly identifiable choroidal neovascularization membrane in the OCT-A scans. In the remaining eyes the identification of a clear CNV membrane was limited at the time of the study due to progressed fibrosis and/or GA following a long-term anti-vascular endothelial growth factor (anti-VEGF) treatment. Seventy eyes (64.8%) were initially fluorescein angiography diagnosed as occult lesions, 27 (25%) as minimally classic, 8 (7.4%) as predominantly classic, and three as retinal angiomatous proliferation. Mean area of CNV was 2.10 ± 3.23 mm^2^ (range 0–24.32 mm^2^). Thirty-five eyes had sparing in the central 1 mm.

### Correlations Between Visual Acuity Parameters and FAF, OCT, and OCT-A Parameters

The correlations are presented for all eyes as well as separately for the better and the worse eyes, respectively, in Table 3. All visual acuity parameters were statistically significantly correlated with the GA parameters measured by FAF when considering all eyes (Table 3).

**Table 3:** Correlations between visual acuity measures, OCT, FAF and OCT-A parameters.

	**BCVA (ETDRS letters)**			**LLVA (ETDRS letters)**			**CS (log units)**			**MRS monocular (wpm)**		
	**All eyes**	**Better eyes**	**Worse eyes**	**All eyes**	**Better eyes**	**Worse eyes**	**All eyes**	**Better eyes**	**Worse eyes**	**All eyes**	**Better eyes**	**Worse eyes**
**FAF**												
Total area of macular GA	−0.634	−0.024	−0.694	−0.643	−0.010	−0.710	−0.766	−0.706	−0.763	−0.661	−0.602	−0.648
Percentage of GA in central 1 mm	−0.651	−0.107	−0.720	−0.644	−0.062	−0.725	−0.693	−0.549	−0.709	−0.688	−0.532	−0.690
**OCT**												
IS/OS interface disruption score in central 1 mm	−0.537	−0.451	−0.548	−0.525	−0.390	−0.584	−0.619	−0.523	−0.548	−0.596	−0.470	−0.632
ELM disruption score in central 1 mm	−0.590	−0.469	−0.572	−0.586	−0.393	−0.555	−0.591	−0.495	−0.572	−0.601	−0.451	−0.697
CRT	−0.423	0.102	−0.436	−0.450	−0.046	−0.457	−0.461	0.054	−0.539	−0.324	0.250	−0.400
PED height in central 1 mm	0.064	−0.119	0.106	0.050	−0.163	0.078	0.026	0.018	0.017	0.142	0.241	0.094
**OCT-A**												
Total area of CNV	−0.558	0.197	−0.640	−0.572	0.078	−0.647	−0.588	0.135	−0.698	−0.416	0.106	−0.502

*Correlations between multimodal imaging parameters and visual acuity parameters for all eyes (n = 108), better and worse eyes (n = 54, respectively): p < 0.001—dark gray filled cells, p ≥ 0.001 to <0.01—medium gray filled cells*.

**Figure 1:**
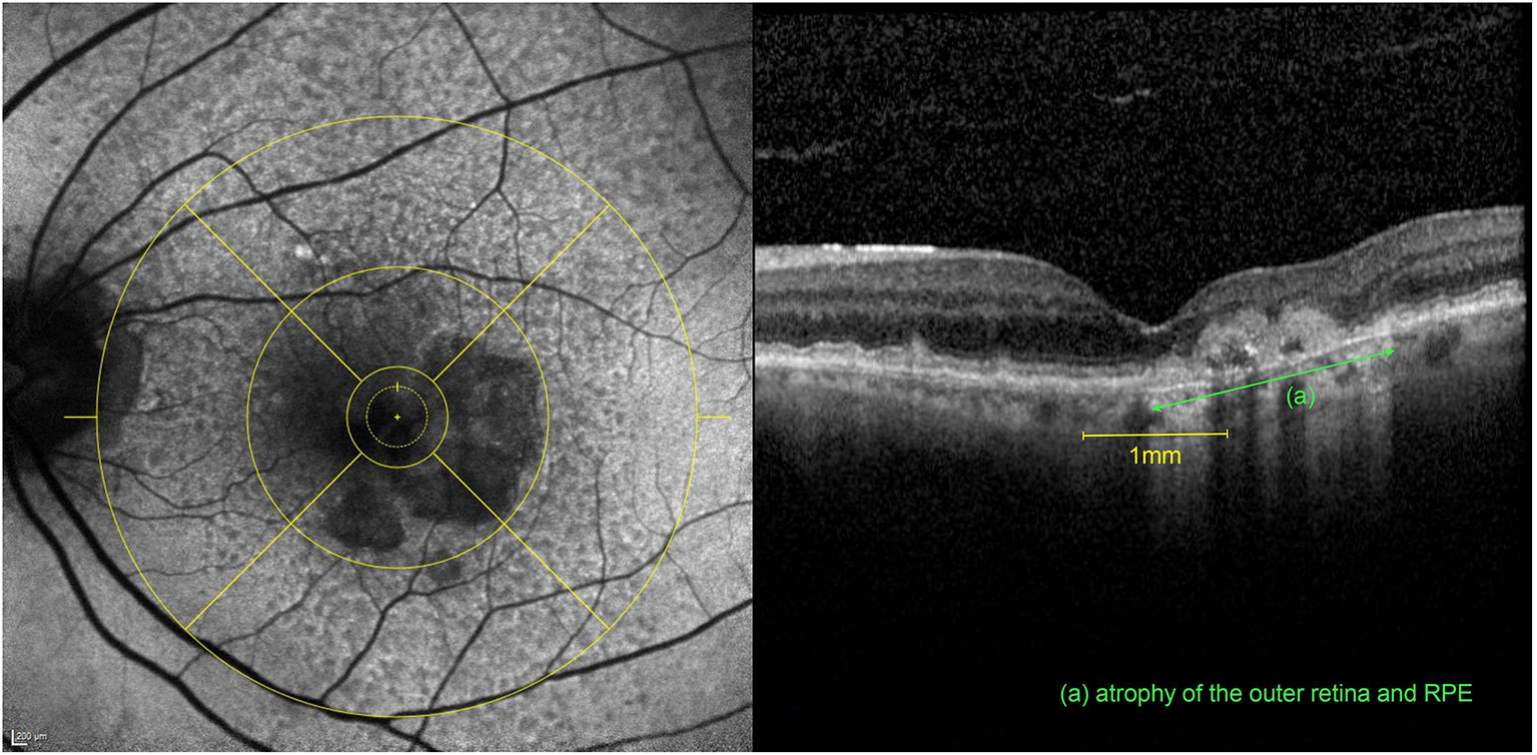
Multimodal imaging of the left eye of an 81-year-old patient after 6 intravitreal aflibercept and 19 ranibizumab injections for nAMD. The patient had a preserved BCVA of 80 letters ETDRS with an already diminished CS (1.20 log units) and monocular MRS (81.28 wpm) (compared to the mean values of all included eyes of 66.7 letters ETDRS, 1.21 log units CS and 96.9 wpm monocular MRS, respectively). **Left:** FAF image with an ETDRS grid overlay centered on the fovea with a horseshoe-shaped area of atrophy involving the central 1mm. **Right:** Corresponding volume OCT scan revealing at the same time, an area of intact anatomic preservation on the left, associated with an atrophy of the outer retina (ELM and IS/OS interface disruption) and the retinal pigment epithelium (RPE) (green) on the right within the fovea.

Strongest correlations were observed between contrast sensitivity and total area of macular GA (*r* = −0.766, *p* < 0.001) as well as percentage of GA in the central 1 mm (*r* = −0.693, *p* < 0.001) for all eyes. Interestingly, in the better eye group BCVA and LLVA did not correlate with the FAF parameters whereas CS and maximum reading speed still exhibited strong correlations. Figure 1 illustrates an eye with GA involving the central 1 mm but leaving an area of anatomic foveal preservation. The patient still had a well-preserved standard ETDRS distance BCVA of 80 letters but already showed a diminished CS and monocular MRS (1.20 log units and 81.28 wpm, respectively). For the correlations of maximum binocular reading speed and morphologic parameters, only the better eyes (*n* = 54) were included since there was no statistically significant difference between maximal binocular reading speed and maximal monocular reading speed of the better eyes. The binocular MRS was moderately correlated with the FAF parameters (*r* = −0.395, *p* = 0.001). Furthermore, significant associations were found between the NEI-VFQ25 near distance subscale and the total area of macular GA in the better eye group (*r* = −0.493, *p* = 0.001).

Considering the OCT parameters strong negative correlations were seen between IS/OS interface and ELM disruption score in the central 1 mm and all of visual acuity parameters except binocular MRS for which correlations were moderate (*p* < 0.001). Compared to the other functional measures, CS showed the strongest correlations with the mentioned OCT parameters with the highest correlation coefficient with the IS/OS interface disruption score when considering all eyes (*r* = −0.619, *p* < 0.001). The correlation coefficients were comparable between the better and the worse eye group although they were superior for CS than BCVA and LLVA in the better eyes. CRT exhibited moderate statistically significant negative correlations with all measures of visual acuity measured monocularly in the worse eyes. The maximal height of pigment epithelial detachment in the central 1 mm was not significantly correlated with visual function. Eyes with reticular pseudodrusen (*n* = 25) did not show a significant difference in BCVA (*p* =0.156), LLVA (*p* = 0.297), CS (*p* = 0.276), or MRS (*p* = 0.875) when compared to eyes without them (*n* = 83).

The total area of CNV exhibited moderate negative correlations with the visual acuity parameters with strongest correlation with contrast sensitivity for all eyes (*r* = −0.588, *p* < 0.001). However, the total area of CNV was not significantly correlated with visual function when including only the better eyes.

### Relation Between Location of Lesions and Visual Acuity Parameters

Table 4 shows the influence of the presence or absence of each lesion component in the different ETDRS subfields on the different visual acuity parameters. The inner subfields of the ETDRS grid were summarized as the central 3 mm ring and the outer subfields as the central 6 mm ring. Comparison of means showed that all visual acuity parameters where significantly worse in the presence of geographic atrophy and subretinal fibrosis in the central 1, 3, and 6 mm ring when considering all eyes. A disrupted IS/OS interface and ELM in the central 3 mm, respectively, 6 mm was associated with a significantly lower CS both in the better and the worse eye group whereas BCVA and LLVA were not significantly changed. Eyes with a CNV in the central 3 mm exhibited a significantly worse CS, LLVA and BCVA considering all eyes. The presence of a PED was not associated with significantly worse visual acuity parameters.

**Table 4:** Relations between location of lesions and visual function.

	***p*****-values comparing presence of lesion and visual acuity parameters (unpaired** ***t*****-test)**											
	**BCVA (ETDRS letters)**			**LLVA (ETDRS letters)**			**CS (log units)**			**MRS monocular (wpm)**		
	**All eyes**	**Better eyes**	**Worse eyes**	**All eyes**	**Better eyes**	**Worse eyes**	**All eyes**	**Better eyes**	**Worse eyes**	**All eyes**	**Better eyes**	**Worse eyes**
**FAF**												
Presence of GA in central 1 mm	0.016	0.285	0.016	0.016	0.572	0.016	0.016	0.015	0.016	0.016	0.048	0.016
Presence of GA in central 3mm	0.016	1	0.016	0.016	0.068	0.016	0.016	0.156	0.016	0.016	0.650	0.016
Presence of GA in central 6mm	0.016	1	0.052	0.015	1	0.039	0.016	0.924	0.015	0.016	1	0.028
**OCT**												
IS/OS interface disruption												
Present in central 3 mm	1	0.285	1	1	0.252	1	1	0.042	1	1	1	1
Present in central 6 mm	1	0.742	0.770	1	1	0.616	0.126	1	0.015	1	1	0.015
ELM disruption												
Present in central 3 mm	1	0.285	1	1	0.252	1	1	0.042	1	1	1	1
Present in central 6 mm	1	1	0.228	1	1	0.192	0.126	1	0.042	1	1	0.015
Subretinal fibrosis												
Present in central 1 mm	0.016	0.016	0.016	0.016	0.016	0.016	0.016	0.016	0.016	0.016	0.182	0.016
Present in central 3 mm	0.016	0.016	0.016	0.016	0.015	0.016	0.016	0.015	0.016	0.016	0.150	0.016
Present in central 6 mm	0.015	1	0.016	0.015	1	0.016	0.015	1	0.016	0.015	1	0.016
PED												
Present in central 1 mm	0.280	1	0.450	0.392	1	0.550	0.364	1	0.672	0.015	1	0.120
Present in central 3 mm	1	1	1	1	1	1	1	1	1	1	1	1
Present in central 6 mm	1	1	1	1	1	1	1	1	1	1	1	1
OCT-A												
Presence of CNV in central 1 mm	0.015	1	0.028	0.015	1	0.028	0.444	1	0.130	1	1	1
Presence of CNV in central 3 mm	0.016	1	0.015	0.016	1	0.015	0.045	1	0.015	0.392	1	0.52
Presence of CNV in central 6 mm	0.943	1	1	1	1	1	1	1	1	1	1	1

*Relations between presence or absence of lesion components in different locations and visual acuity parameters for all eyes (n = 108), better and worse eyes (n = 54, respectively), p-values adjusted for multiple comparisons with the Bonferroni-Holm adjustment. p ≥ 0.01 to 0.05—light gray filled cells*.

### Multiple Regression Analysis

Multiple regression analysis run each time with a visual function parameter as the dependent variable, yielded values of adjusted R^2^ of 0.707 for contrast sensitivity, 0.596 for BCVA, 0.588 for LLVA and 0.591 for monocular MRS when all eyes were included. The model with contrast sensitivity as the dependant variable with the highest adjusted R^2^ is summarized in Table 5. This model selected the total area of macular GA, the IS/OS interface disruption score, the presence of subretinal fibrosis in the central 1 mm and CRT as significant independent parameters. Therefore, these factors explain ~71% of the variation of contrast sensitivity.

**Table 5:** Multiple regression analysis of contrast sensitivity and morphologic parameters.

**Independent variables**	**Adjusted R^**2**^**	**Beta coefficient of regression**	***p*-value**
All eyes (*n* = 108)	0.707		
Total area of macular GA		−0.035	<0.001
IS/OS interface disruption score		−0.088	0.004
Subretinal fibrosis in central 1mm		−0.191	0.030
CRT		−0.001	<0.001
Better eyes (*n* = 54)	0.586		
Total area of macular GA		−0.042	<0.001
IS/OS interface disruption score		−0.100	0.001
Worse eyes (*n* = 54)	0.772		
Total area of macular GA		−0.041	<0.001
IS/OS interface disruption score		−0.112	<0.001
CRT		−0.001	0.011

When subdividing into two groups depending on the status of the eye (better vs. worse), results indicated a higher coefficient of determination for the worse eyes (0.772 vs. 0.586). Furthermore, only the total area of macular GA, the IS/OS junction line disruption score and CRT (only for the worse eyes) were selected as significant independent variables.

## Discussion

Several studies have reported that anatomic parameters such as CNV area and diameter do not adequately explain changes in distance visual acuity in nAMD (23, 26). Yet, the majority of those studies did not include a multitude of morphologic parameters graded on the basis of the ETDRS grid subfields using latest image acquisition devices such as SD-OCT and OCT-A and did not evaluate different measures of visual function.

Our results suggest that contrast sensitivity (CS) exhibits the most consistent correlations with many of the anatomic parameters compared to the other assessed measures of vision. Likewise, Ghoshal et al. correlated different functional measures and retinal morphology and found CS to be associated with a few OCT parameters including thickness and volume of RPE and Bruch membrane (32). In our study, although significant correlations were observed for all measures of visual function, CS exhibited the strongest correlation coefficients for all the evaluated morphologic parameters and yielded the highest coefficient of determination in multiple regression analysis.

With regards to the component of the lesions, in our study the area of atrophy and the presence of subretinal fibrosis in the central 1 mm were selected as independent variables explaining the variation in CS for all eyes. In addition, their presence in all of the ETDRS subfields was significantly correlated with CS (for the better eye group only in the central 1 mm). Their influence on visual function in general is not surprising as it is in accordance with several other studies (23, 33). Subretinal fibrosis as the end stage of nAMD leads to a destruction of the overlying photoreceptors; on the one hand by interfering with the metabolic exchange with the RPE and on the other hand by a direct toxic effect (34). Likewise, subretinal fibrosis and atrophy go along with a destruction of the outer retina and the RPE resulting in decreased visual function. Similarly, Keane et al. reported strongest correlations between a decreased CS and an increased subretinal tissue volume for newly diagnosed nAMD patients explaining 24% of the variation in CS at baseline (24).

In our study both the total area of geographic atrophy (GA) as defined by hypoautofluorescence in autofluorescence imaging as well as the percentage of GA in the central 1 mm were strongly associated with CS and other measures of visual function. Accordingly, Ooto et al. reported a negative correlation between CS and size of confluent hypoautofluorescence as well as its involvement of the fovea in dry AMD (35). Despite the additional presence of other components in nAMD our results suggest that GA is a major factor influencing visual function in nAMD, too. Furthermore, we identified the percentage of GA in the central 1 mm as a significant influencing factor which has been previously described to better correlate with distance visual acuity than total GA size (22). In addition, it can serve as a better tool to predict visual impairment over time than the simply binary grading of foveal-sparing status. Furthermore, in our study the presence of GA in the central 3 and 6 mm had a significant impact on visual function. Correspondingly, Sayegh et al. reported that progression of GA depends on the distance to the fovea and argue for a grading of sparing in the central 3 mm as it correlates with both atrophy progression and visual acuity (36).

With newer OCT devices, the visualization of the degeneration of the photoreceptors is thought to be visible by a disruption of ELM and the IS/OS interface which may be present not only in atrophic areas but also in the junctional zones (37). In our study, the ELM and IS/OS interface disruption score exhibited strongest correlations with CS and a disruption of ELM and IS/OS interface in the central 3 mm (better eye group) and the central 6 mm (worse eye group) was significantly associated only with CS but not the other measures of vision. Whereas, the size of the ellipsoid zone defect has been shown to correlate with microperimetry scores (35), there are currently no studies investigating the relation between ELM and IS/OS interface disruption and CS in anti-VEGF treated nAMD patients.

Although distance visual acuity is widely used in clinical trials, several studies documented that it correlated less with morphological features in nAMD than for example near visual acuity (23, 26), indicating that standard BCVA at 4 m does not adequately correlate with foveal impairment in nAMD. In the present study, correlation coefficients were also generally weaker compared to CS. We observed a similar significant but moderate correlation between central retinal thickness (CRT) and ETDRS BCVA as well as CS when considering all eyes. Retinal thickening associated with intraretinal fluid exudation is commonly observed in nAMD. Although a reduction in CRT after anti-VEGF treatment has been shown to moderately correlate with long-term visual outcomes, many studies failed to detect stronger correlations between CRT and visual function (24, 38, 39). Similarly, our results may be reflective of the heterogeneous morphology in AMD where reduced retinal thickness in advanced atrophic lesions is associated with severe vision loss. Further, in our study the total area of CNV as evaluated on OCT-A was moderately correlated with visual function in the worse eyes and its presence in the central 1 and 3 mm showed a highly significant association with ETDRS visual acuity. In other studies based on angiographic measurements, these correlations were weaker and inconsistent (23, 26), probably due to the fact that our study cohort had already experienced a long disease duration so that the CNV had often resulted in a considerable amount of atrophy or fibrosis within the macula. With growth of the CNV below the RPE, the formation of a fibrovascular PED may occur which is often accompanied by leakage or hemorrhage. Our results did not indicate a correlation between the presence or the height of PED and ETDRS BCVA which is consistent with findings of other studies (24, 40). This lack of association is not surprising as even large PED may go along with functioning overlying photoreceptors. Furthermore, we did not characterize the PED subtypes, thus a possible correlation dependent on the structural characteristics of the PED remains unclear.

Whereas, LLVA has been shown to capture visual impairment in nAMD better than standard distance acuity (41), there is currently limited data correlating systematically LLVA and various morphologic characteristics. The Chroma and Spectri studies reported a weak negative correlation between LLVA and GA lesion size at baseline and week 48 in bilateral GA in dry AMD (42). Further, patients with foveal involvement already at baseline showed less decline in LLVA but not the other functional measures which reflects its relevance as a measure of foveal cone function in early disease stages in the absence of foveal lesions. In the present study LLVA showed globally similar correlations with retinal morphology than standard distance acuity, most likely due to their high intercorrelation level.

While reading speed is known to be significantly reduced in eyes with nAMD (9), in our study, correlations between the binocular MRS and the morphologic impairment of the better eye were globally weaker and inconsistent. This was probably due to the fact that the influence of the fellow eye was not taken into account. Since reading is performed best with central vision, monocular reading which prevents the compensation of a monocular central field loss by the fellow eye, is severely compromised in nAMD, possibly resulting in weaker correlations for the monocular MRS. Nevertheless, in our study monocular MRS exhibited stronger correlation coefficients with the autofluorescence parameters and ELM and IS/OS interface disruption score than standard BCVA. Reviewing the literature, reading speed has been described to correlate significantly with the size of GA (34, 42), central retinal thickness (24) as well as the foveal involvement of atrophy and subretinal fibrosis (23). Our results correspond to these findings with significant relations between monocular MRS and the presence of GA and subretinal fibrosis in the central 1, 3, and 6 mm considering all eyes. Especially the size of the foveal spared area seems to influence MRS as it has been shown that a minimal spared central area is required for fluent reading, otherwise the characters would not “fit” in the preserved space (43). Further, the Chroma and Spectri studies found MRS to be best correlated with progression of GA size compared to other functional measures in bilateral GA in dry AMD (42). Likewise, in our study strongest correlations for the monocular MRS were observed with the percentage of GA in the central 1 mm.

Analysis with two subgroups (better vs. worse eyes) revealed similar strong correlation coefficients between CS and the GA parameters as well as the disruption of the IS/OS interface and ELM independently of the status of the eye. However, standard BCVA was not reflective of the atrophic lesions in the better eyes. Hence, CS could serve as an early biomarker of impaired visual function especially in early disease stages and therefore be used even in the better eyes of patients with bilateral nAMD. Generally, the correlation coefficients were lower in the better eye than in the worse eye group which is consistent with findings in the literature (27). Possibly, the better eyes performed better than expected considering their morphologic lesions, since the subjects largely depended on it due to the impairment of the fellow eye.

To the best of our knowledge, there are no studies so far that systemically correlated a multitude of morphologic parameters graded on FAF, SD-OCT and SS-OCT-A with several measures of visual function of subjects with bilateral nAMD. The performance of a standardized visual assessment protocol under the same conditions and the homogenous study population contribute to the strengths of our study. Further standardized image evaluation protocols were used for FAF, SD-OCT and SS-OCT-A analyses. Limitations of this study included its cross-sectional design and relatively small study population. Unfortunately, due to a lengthy disease course a reliable statement about the activity status based on shape/branching pattern/anastomoses/morphology of vessel termini/perilesional halo was not always possible in the cross sectional OCTA exam. Furthermore, the LLVA testing protocol of our clinic did not include the use of a KODAK filter, thus potentially precluding a bigger low luminance deficit. Our results should be validated in larger cohorts evaluating how the progression of morphological impairment correlates with visual function over time in order to investigate its predictive value on future visual acuity change.

To summarize, in this study visual function did not depend on an unique anatomic parameter indicating that in nAMD which does not present as a single homogenous morphologic entity, the extent, composition and size of the lesions taken together account for the associated visual impairment. This study suggests that in bilateral nAMD contrast sensitivity is better correlated with anatomic characteristics than other functional visual measures including standard ETDRS BCVA with strongest correlations with total area of macular GA and IS/OS interface disruption score. These correlations were consistent even in the better eyes of a patient contrary to standard BCVA, thus CS may serve as a better biomarker in early disease stages. Furthermore, in comparison to the other assessed visual function measures, CS has been shown to best correlate with vision-related quality of life as reflected by the NEI-VFQ25 distance and near distance score (21). Thus, given the more consistent correlation with morphologic characteristics, the incorporation of CS as a surrogate endpoint in clinical trials may lead to improved accuracy. Furthermore, it may be used as a standard diagnostic tool in clinical practice to better reflect the patient's individual visual impairment.

## Data Availability Statement

The raw data supporting the conclusions of this article will be made available by the authors, upon personal request.

## Ethics Statement

The studies involving human participants were reviewed and approved by Ethikkommission Nordwest- und Zentralschweiz. The patients/participants provided their written informed consent to participate in this study.

## Author Contributions

LH and PR: acquisition, interpretation of data, and statistics and writing of manuscript. M-MG: interpretation of data. KH: design of the study, acquisition, interpretation of data, supervision, and writing of manuscript. All authors critically reviewed the manuscript and approved the final version.

## Conflict of Interest

The authors declare that the research was conducted in the absence of any commercial or financial relationships that could be construed as a potential conflict of interest.
